# Noradrenergic Add-on Therapy with Extended-Release Guanfacine in Alzheimer’s Disease (NorAD): study protocol for a randomised clinical trial and COVID-19 amendments

**DOI:** 10.1186/s13063-022-06190-3

**Published:** 2022-08-01

**Authors:** Karen Hoang, Hilary Watt, Mara Golemme, Richard J. Perry, Craig Ritchie, Danielle Wilson, James Pickett, Chris Fox, Robert Howard, Paresh A. Malhotra

**Affiliations:** 1 Department of Brain Sciences, Imperial College London, London, UK; 2 Imperial College Healthcare NHS Trust, London, UK; 3 Department of Public Health and Primary Care, Imperial College London, London, UK; 4 UK Dementia Research Institute Care Research and Technology Centre, Imperial College London and the University of Surrey, London, UK; 5 Edinburgh Dementia Prevention and Centre for Clinical Brain Sciences, Edinburgh Medical School, University of Edinburgh, Edinburgh, UK; 6 Norwich Medical School, University of East Anglia, Norfolk, UK; 7 Division of Psychiatry, University College London, Maple House 149 Tottenham Court Road, London, W1T 7NF UK

**Keywords:** Alzheimer’s disease, Guanfacine, Add-on therapy, COVID-19

## Abstract

**Background:**

Guanfacine is a α2A adrenergic receptor agonist approved for treating attention deficit hyperactivity disorder (ADHD). It is thought to act via postsynaptic receptors in the prefrontal cortex, modulating executive functions including the regulation of attention. Attention is affected early in Alzheimer’s disease (AD), and this may relate to pathological changes within the locus coeruleus, the main source of noradrenergic pathways within the brain. Given that cholinergic pathways, also involved in attention, are disrupted in AD, the combination of noradrenergic and cholinergic treatments may have a synergistic effect on symptomatic AD. The primary objective of the NorAD trial is to evaluate the change in cognition with 12 weeks of treatment of extended-release guanfacine (GXR) against a placebo as a combination therapy with cholinesterase inhibitors in participants with mild to moderate Alzheimer’s disease.

**Methods/design:**

NorAD is a 3-month, single-centre, randomised, double-blind, placebo-controlled, phase III trial of extended-release guanfacine (GXR) in participants with mild to moderate Alzheimer’s disease. A total of 160 participants will be randomised to receive either daily guanfacine or placebo in combination with approved cholinesterase treatment for 12 weeks. The primary outcome is the change in cognition, as measured by the Alzheimer’s Disease Assessment Scale-Cognitive Subscale (ADAS-Cog), from baseline to follow-up in the treatment group compared to the placebo group. Secondary outcomes include the change in additional cognitive measures of attention (Tests of Attention: Trails A and B, digit-symbol substitution, Test of Everyday Attention and CANTAB-RVP), neuropsychiatric symptoms (Neuropsychiatric Inventory), caregiver burden (Zarit Burden Interview) and activities of daily living (Alzheimer’s Disease Co-operative Study – Activities of Daily Living Inventory). From July 2020, observation of change following cessation of treatment is also being assessed.

**Discussion:**

There is strong evidence for early noradrenergic dysfunction in Alzheimer’s disease. The NorAD trial aims to determine whether guanfacine, a noradrenergic alpha-2 agonist, improves attention and cognition when used in addition to standard cholinergic treatment.

**Trial registration:**

ClinicalTrials.govNCT03116126. Registered on 14 April 2017

EudraCT: 2016-002598-36

## Administrative information

**Table:** 

Title {1}	Noradrenergic Add-on Therapy with Extended-Release Guanfacine in Alzheimer’s Disease (NorAD): study protocol for a randomised clinical trial and COVID-19 amendments
Trial registration {2a and 2b}.	ClinicalTrials.gov, NCT03116126. Registration 14 April 2017 EudraCT: 2016-002598-36
Protocol version {3}	15 Jun 2016 v1.0 – Original 26 Aug 2016 v.1.1 – Amendment stating the stratification of patients into mild and moderate AD 16 May 2017 v1.2 – Amendment minor changes and corrections to protocol following TSC (Trial Steering Committee) meeting to clarify statements 26 Sep 2018 v1.3 – Amendment minor changes updating contact information and references 19 Mar 2020 v1.4 – Amendment to include special COVID measures addressing March 2020 COVID-related research suspension 08 Jun 2020 v1.5 – Amendment to resume trial activities post-COVID research suspension. The addition of the ADCS-CGIC and audio recording of cognitive assessments.
Funding {4}	Active drug and placebo for the trial are provided through by an Investigator-Initiated Research grant (IIR-GBR-000792) from Takeda (formerly Shire) Pharmaceuticals and the trial is funded by the UK National Institute for Health Research (NIHR) through the Research for Patient Benefit (RfPB) Programme (Grant reference: PB-PG-0214-33098). The design, management, analysis and reporting of the study are independent of manufacturer of the drug and placebo. The views expressed are those of the author(s) and not necessarily those of the NIHR or Department of Health and Social Care. Additional support for the trial has been provided by Imperial College Healthcare NHS Trust and the NIHR Biomedical Research Centre at Imperial College London.
Author details {5a}	Karen Hoang ^1,2^, Hilary Watt ^3^, Mara Golemme ^4^, Richard J. Perry ^1,2^, Craig Ritchie ^5^, Danielle Wilson ^4^, James Pickett, Chris Fox ^6^, Robert Howard ^7^, Paresh A. Malhotra ^1,2,4^ 1. Department of Brain Sciences, Imperial College London, United Kingdom 2. Imperial College Healthcare NHS Trust, London, United Kingdom 3. Department of Public Health and Primary Care, Imperial College London, United Kingdom 4. UK Dementia Research Institute Care Research and Technology Centre, Imperial College London and the University of Surrey, United Kingdom 5. Edinburgh Dementia Prevention and Centre for Clinical Brain Sciences, Edinburgh Medical School, University of Edinburgh, Edinburgh, United Kingdom 6. Norwich Medical School, University of East Anglia, Norfolk, United Kingdom 7. Division of Psychiatry, University College London, Maple House 149 Tottenham Court Road, London W1T 7NF, United Kingdom *PAM* conceived the study. *PAM*, *RJP*, *CR*, *DW*, *JP*, *CF* and *RH* initiated the study design and *KH* and *MG* with implementation. *PAM* is the grant holder. *HW* provided statistical expertise in the clinical trial design and is conducting the primary statistical analysis. All authors contributed to the refinement of the study protocol and approved the final manuscript.
Name and contact information for the trial sponsor {5b}	**Trial Sponsor:** Imperial College London **Sponsor’s Reference:** 16IC3372 **Contact name:** Research Governance and Integrity Team (RGIT) **Address:** Imperial College Healthcare NHS Trust, Norfolk Palace Road, London, W1 2PG **Telephone:** 02033110204 **Email:** rgit.ctimp.team@imperial.ac.uk
Role of sponsor {5c}	The sponsor and funding source had no role in the design of this study and will not have any role during its execution, analysis, interpretation of the data or decision to submit results.

## Background

Alzheimer’s disease (AD), the most common cause of dementia worldwide, is associated with the disruption of multiple neurotransmitter systems [1]. In the last two decades, there has been a strong focus on developing medications that reverse or slow pathological progression, even though there remains a clear need for effective symptomatic treatment. Current approved medication has predominantly targeted the cholinergic system. Acetylcholinesterase inhibitors (AChEIs) were developed based on the well-established association between cholinergic dysfunction with memory symptoms and observed degeneration of cholinergic pathways in the brainstem [2]. Three cholinesterase inhibitors (ChEIs) donepezil, galantamine and rivastigmine are currently recommended as a treatment for patients with mild to moderate AD dementia. A further approved treatment is the less commonly prescribed NMDA receptor antagonist, memantine, which is recommended for use in moderate and severe disease [3]. However, these medications have a very limited impact on cognitive function as well as indices assessing activities of daily living [4].

The current evidence suggests that after the initial amnestic stage in typical AD, attention is one of the earliest cognitive domains to be affected [5]. Attentional processes are closely linked to brainstem arousal systems, particularly the locus coeruleus (LC), a noradrenergic/norepinephrine (NE) pontine nucleus with projections throughout the cerebral cortex [6]. Remarkably, animal models and post-mortem human studies have indicated that tau pathology appears very early in the LC, and it has been suggested that LC dysfunction may contribute to the initiation, progression and severity of the disease [7]. In addition to its direct role in attention and arousal, the LC may also play a key part in memory [8] and to the neuropsychiatric symptoms often encountered in AD [9].

Indeed, researchers have suggested that there is a role for noradrenergic therapy in AD, particularly in individuals who have impaired attention [10, 11]. Further, there is some evidence that NE pathways are important in mediating cognitive reserve in AD, providing additional support for the exploration of NE agents as a potential therapy [12]. Importantly, it has also been proposed there is an interaction between acetylcholine (ACh) and norepinephrine (NE) in increasing vigilance and attention in associative learning processes in memory [13], suggesting that there may be a synergistic effect between cholinergic and noradrenergic agents. Therefore, there is a strong theoretical underpinning for using noradrenergic agents in combination with cholinesterase inhibitors.

The majority of previous studies of combined noradrenergic/cholinergic therapy in AD have tended to be underpowered or of limited duration [14]. For instance, Mohs and colleagues assessed the effects of combining a noradrenergic agent with current standard cholinergic therapy in a trial of 92 patients with symptomatic mild-moderate AD [15]. They found no additional effect of atomoxetine, a selective noradrenergic re-uptake blocker, on cognition in comparison with placebo but noted that if their analysis had been further powered, a clearer change in functional decline might have been observed. Further, the authors noted that measures such as the Alzheimer’s Disease Co-operative Study – Activities of Daily Living Inventory (ADCS-ADL) might offer the most sensitive means of detecting the effects of add-on therapy.

One promising agent in this context is an alpha-2A agonist, guanfacine [16]. Originally developed as an antihypertensive, guanfacine is licenced as a non-stimulant therapy in childhood attention deficit hyperactivity disorder (ADHD), with an extended-release formulation approved for once-daily dosing. Guanfacine acts via postsynaptic receptors in the prefrontal cortex [17, 18], and there is also a suggestion from animal models that it may rescue neuronal damage in tandem with improving cognition [19]. In primates as well as healthy humans, guanfacine has been shown to affect aspects of attention and working memory [17], although a recent study found no effect of 3 months of treatment with a low dose of extended-release guanfacine on attention or cognitive function in healthy elderly controls [20].

Guanfacine has been evaluated as a cognitive enhancer in neurological conditions other than ADHD. In small proof-of-principle studies, it has been shown to be tolerated and to enhance attention in patients with stroke [21] and neuroinflammatory disease [22, 23]. A number of historical studies have examined the potential role of guanfacine as a single agent in patients with cognitive impairment, including Parkinson’s disease dementia and Alzheimer’s disease [24, 25]. Although guanfacine did appear to modulate cognition in the former group, studies in AD did not demonstrate any effect [24, 26, 27]. However, it should be noted that these studies were underpowered and also employed a lower dose than that used in ADHD as well as the more recent studies in other disease groups cited above. Furthermore, they examined guanfacine as a single agent, rather than in combination with cholinergic treatments.

In the NorAD trial, funded by the United Kingdom National Institute of Health Research (Research for Patient Benefit Grant PB-PG-0214-33098), we aim to test the efficacy of 2 mg extended-release guanfacine (GXR) against placebo as an add-on therapy in combination with AChEI in patients with mild to moderate Alzheimer’s disease. The primary outcome measure is cognition measured by the Alzheimer’s Disease Assessment Scale – Cognitive Subscale (ADAS-Cog). The secondary outcomes are standard neuropsychological and computerised measures of attention, neuropsychiatric symptoms, caregiver burden, activities of daily living and medication compliance.

## Methods and design

### Study design

NorAD is a 12-week, single-centre, randomised, parallel-group, double-blind, placebo-controlled phase III trial in participants with a clinical diagnosis of mild to moderate AD, who are on a stable dose of ChEI. Eligible participants are randomised in a 1:1 ratio to receive guanfacine or placebo, with randomisation stratified into mild (MMSE 21–30) and moderate (MMSE 10–20) AD. A total of 160 participants (80 on GXR + ChEI and 80 on placebo + ChEI) will be recruited from sites across the UK, from local Memory Clinics at Imperial College Healthcare NHS Trust to other participant identification centres (PIC), North West London Clinical Research Network (CRN), the Primary Care network and the NIHR Join Dementia Research (JDR) registry (https://www.joindementiaresearch.nihr.ac.uk/). Participants are treated for a total of 12 weeks starting treatment with a daily dose of 1 mg (guanfacine or placebo) for the first week, followed by 2 mg daily for the remaining 11 weeks. Those individuals who do not tolerate the increased dose of active drug or placebo, return to the lower dose (1 mg) for the remainder of the trial.

### Outcome measures

#### Primary outcome

The following is the primary outcome:

- Cognition as measured by the Alzheimer’s Disease Assessment Scale – Cognitive subscale (ADAS-Cog) [28] completed at week 0, week 4 and week 12.

#### Secondary outcome

The following are the secondary outcomes:

- Tests of Attention: Trails A and B [29], digit-symbol substitution [30], Test of Everyday Attention [31] (Map Search and Elevator Counting with Distraction) and CANTAB-RVP completed at week 0, week 4 and week 12.
- Carer Input and Activities of Daily Living: Neuropsychiatric Inventory (NPI) [32], Zarit Burden Interview (22 items) [33] Alzheimer’s Disease Co-operative Study – Activities of Daily Living Inventory (ADCS-ADL) [34] completed at week 0, week 4 and week 12.
- Toxicity and tolerability: blood pressure taken at week 0, week 1, week 4, week 8 and week 12; Epworth Sleepiness Scale [35] completed at week 0, week 1, week 4, week 8 and week 12; and common side effects of guanfacine (dry mouth, sedation, fatigue [18]) taken at week 1, week 2, week 4, week 8, week 12 and week 13.
- Compliance: unused dose counts at each visit: significant non-compliance will be defined as the omission of more than 2 doses per week (on average) since the previous visit taken at week 1, week 4, week 8 and week 12.

#### Global Clinical Impression Index

The following is the Global Clinical Impression Index:

- Observation of change following cessation of treatment as measured by Alzheimer’s Disease Co-operative Study – Clinical Global Impression of Change (ADCS-CGIC) [36]. The ADCS-CGIC was implemented from July 2020 following observation that some patients/carers reported perceived changes following cessation of treatment carried out at week 12 and week 13.

### Eligibility criteria

The inclusion and exclusion criteria are presented in Table 1.

**Table 1:** Inclusion and exclusion criteria

**Inclusion criteria**	
1. Outpatient with the capacity to give informed consent 2. Age 45 years or older 3. Diagnosis of probable AD according to the National Institute of Neurological and Communicative Disorder and Stroke and Alzheimer’s Disease and Related Disorders Association (NINCDS-ADRDA) criteria 4. Mini-Mental State Examination (MMSE) score of 10–30 5. An identified individual who can act as a reliable study partner to accompany the participant to all visits (face-to-face visits and telephone contact) and provide input into rating scales 6. On a stable dose of donepezil, galantamine or rivastigmine for the preceding 12 weeks 7. Fluency in English	
**Exclusion criteria**	
1. Labile blood pressure or new antihypertensive medication started within 3 weeks 2. Severe coronary insufficiency or myocardial infarction in the previous 6 months 3. History of unexplained syncope within the preceding 12 months 4. Cardiac conduction block 5. Severe hepatic impairment (ALT > 120, ALP > 390 and total bilirubin > 60) 6. Severe renal impairment (eGFR < 40) 7. Treatment with medications known to potentiate guanfacine’s hypotensive effects or cause arrhythmia (antipsychotics including sultopride, chlorpromazine, thioridazine, amisulpiride, sulpiride, haloperidol), moxifloxacin, baclofen, verapamil, quinidine, hydroquinidine, dispyramide, amiodarone, dofetilide, ibutilide, sotalol, pimazide, bepridil, casipride, diphemanil, erythromycin, halofantrine, pentamidine, sparfloxacin, vincamine, alfuzosin, prazosin, terazosin, tamsulosin and amifostine. Treatment with contradictions to guanfacine, see Appendix 1. 8. Weight under 45 kg (in order to ensure that an excessive dose per body weight is not used in the study) 9. Pregnancy (pre-menopausal women will only be entered into the study if they are surgically sterile or using effective birth control methods: these are abstinence for the period of the study, intrauterine contraception/device, male sexual partners with vasectomy) 10. History of severe chronic obstructive pulmonary disease (COPD) and/or asthma*	

^*^This exclusion criterion was added in August 2020 to reduce potential risk related to COVID-19

### Sample size

The participant number of 160 was determined via a power calculation, intended to achieve 90% power with a 5% significance level using the ADAS-Cog as the primary outcome measure, allowing for a loss to follow-up of 20% over the study period. To detect a mean difference of 3 on ADAS-Cog scores between the groups with an estimated standard deviation (SD) of changes in the ADAS-Cog of 6, we will recruit a total of 160 patients (split into the two groups). We will, in practice, analyse follow-up ADAS-Cog, adjusting for baseline ADAS-Cog score (rather than analysing changes), since adjustment for covariates (including baseline values) is known to increase statistical power (from 80% to a median of 93% in a review of 12 outcomes from 8 studies; because the sample size calculation ignored this baseline adjustment, it was powered to achieve 80% power on the unadjusted analysis [37]. The mean difference of 3 on ADAS-Cog corresponds to the approximate mean change found on cholinesterase inhibitors in AD, which is considered the minimum clinically important difference required to recommend the use of this additional drug. The estimated SD of 6 is based on the mean of the SDs from studies on Alzheimer’s disease, with similar follow-up times, reported in a systematic review by Raina and colleagues [38].

### Study medication

Guanfacine, at doses up to 7 mg, has been approved in several countries, including EU, Canada and the USA (under the trade name Intuniv®), for the treatment of attention deficit hyperactivity disorder (ADHD). In NorAD, participants start with a daily oral dose of 1 mg guanfacine or placebo. The dose will be increased to 2 mg at week 1 if the lower dose is tolerated. The 2 mg dose is based on studies with healthy adults and data obtained in work with elderly individuals with attentional deficits caused by stroke who were treated with standard preparation guanfacine [21] as well a single adult with neuroinflammatory disease [23]. This dose led to clinical improvement in the published reports and caused no side effects in any of these studies. The dose is lower than the maximum dose used for hypertension or ADHD.

As an antihypertensive agent, guanfacine stimulates alpha-2A adrenergic receptors, reducing sympathetic nerve impulses from the vasomotor centre to the heart and blood vessels. However, it has been shown to be well tolerated at significantly higher doses in elderly and paediatric populations and is safe to use in patients with heart failure, renal dysfunction and diabetes [39–41]. Patients with cardiac conduction block are excluded from the NorAD trial. As the extended-release formulation will be used for the current study, peak concentrations will not exceed those for the same dose of standard preparation guanfacine.

### Drug provision

Investigative Medicinal Product (IMP) and placebo are being provided by Takeda (formerly Shire) Pharmaceuticals through an investigator-led grant.

### Randomisation

Participants are randomised to receive active drug or placebo with a 1:1 allocation ratio using the InForm 6.0 trial software (Oracle Health Sciences), which automatically allocates a randomisation code to a participant. Stratification is according to the severity of Alzheimer’s disease based on MMSE scores (moderate 10-20 and mild 21–30). The research team are responsible for randomising the NorAD participants, and the Imperial College Healthcare NHS Trust Pharmacy is responsible for storing and dispensing IMP and placebo.

### Trial assessments

#### Study assessments

Between January 2019 and March 2020, all participants followed the assessments outlined in Table 2. At the week 2 (W2) visit, a telephone call is to check for any adverse events. At the week 12 (W12) visit, final assessments are carried out, and unused study medication is collected. One week after cessation of treatment, there is a final follow-up telephone call (W13). The neuropsychological assessments are completed by a trained rater, an individual trained specifically by a trained dementia research coordinator to conduct the outcome assessments at the research site.

**Table 2:** NorAD trial schedule of visits

Procedures (following pre-screening visit)	Screening visit	0 (W0)	1 (W1)	2 (T/C) (W2)	3 (W4)	4 (W8)	5 (W12)	6 (T/C) (W13)
MMSE	X	X					X	
ECG	X	X	X					
U&E, LFT	X							
HR, s/s BP	X	X	X		X	X	X	
≥ 3 months treatment with ChEI	X	X						
Consent	X	X						
Medical history	X							
Inclusion/exclusion	X	X						
Randomisation		X						
ADAS-Cog		X			X		X	
Trails A and B		X			X		X	
Digit-symbol substitution		X			X		X	
TEA (map search and elevator counting)		X			X		X	
CANTAB-RVP		X			X		X	
NPI		X			X		X	
ZBI		X			X		X	
ADCS-ADL		X			X		X	
ESS		X	X		X	X	X	
SEQ			X	X	X	X	X	X
AE/CM			X	X	X	X	X	X
Unused dose counts			X		X	X	X	
Dispensing		X	X		X	X		

There is a ± 3-day window for each assessment

*ADAS-Cog* Alzheimer’s Disease Assessment Scale-Cognitive Subscale, *ADCS-ADL* Alzheimer’s Disease Co-operative Study - Activities of Daily Living Inventory, *AE/CM* adverse event/concomitant medication, *CANTAB-RVP* Cambridge Neuropsychological Automated Battery-Rapid Visual Information Processing, *ChEI* cholinesterase inhibitor, *ECG* electrocardiogram, *ESS* Epworth Sleepiness Scale, *HR* heart rate, *LFT* liver function test, *MMSE* Mini-Mental State Examination, *NPI* Neuropsychiatric Inventory, *s/s BP* sitting and standing blood pressure, *SEQ* Side-Effect Questionnaire, *TEA* Test of Attention, *U&E* urea and electrolytes, *W* Week, *ZBI* Zarit Burden Interview

#### Progress and COVID-19-related changes

The NorAD trial commenced in January 2019, with 48 patients randomised by March 2020. At that point, due to UK-wide lockdown and COVID-19-related research suspension and following Imperial College Healthcare NHS Trust (ICHT) guidance, NorAD suspended recruitment and all visits at the trial site. Those patients who commenced treatment between 01 January 2020 and 13 March 2020 continued treatment until the date of their next planned visit at which point a telephone assessment was conducted by a trained rater in place of attending at the trial site. The telephone assessment included a shorter version of the assessment battery but did not include any cognitive instruments or vital signs measurements (it should be noted that this was not considered a safety risk as treatment was discontinued at the time of the telephone visit). Telephone assessment consisted of completing safety questions (Adverse Event/Concomitant Medication, Side-Effect Questionnaire, Epworth Sleepiness Scale) and carer questionnaires (Neuropsychiatric Inventory, Alzheimer’s Disease co-operative Study – Activities of Daily Living Inventory, Zarit Burden Interview) where appropriate. Twelve patients prematurely ended trial participation. Of these, 7 had reached the week 4 (W4) visit and therefore had attended their second assessment with outcome data. Five participants ended participation before this stage, with no outcome data available.

#### Trial restart post-COVID-19

NorAD was restarted in August 2020 according to the NIHR restart framework for clinical research (https://www.nihr.ac.uk/documents/restart-framework/24886). Those participants that had previously commenced treatment but not reached the week 4 visit were eligible to restart participation (using the same randomisation code) to complete a full 12 weeks of treatment (from the date of recommencement). Three of those patients have successfully restarted, and the other 2 are considered withdrawn.

Recruitment resumed using local safety measures and a revised set of assessments to minimise COVID risk from multiple in-person attendances. These were approved as a protocol amendment by the Research Ethics Committee prior to the trial restart. Revisions included the week 8 (W8) visit being converted to a telephone call to check for any adverse events rather than an in-person visit. This was not considered a safety risk as at W8 patients have been on the same dose of treatment for 7 weeks, and to date, there have been no reports of new side effects or safety issues at this time point. In addition, following the restart, the week 12 (W12) visit includes the Alzheimer’s Disease Co-operative Study – Clinical Global Impression of Change (ADCS-CGIC) with the final follow-up telephone call (W13) including part 2 of the ADCS-CGIC.

#### Withdrawals and unblinding

Participants who drop out of the NorAD study are recorded as withdrawn. Participants may withdraw consent to participate in the trial and will no longer receive the study treatment or undergo assessments. The chief investigator may withdraw a participant because of clinical factors, discontinuation of the study treatment or a change in circumstances. A “lost to follow-up” would be no news of a participant after every effort to contact the participant or informant. In all cases, the investigator suggests the participant attends a study end visit, and substantial effort will be made to obtain follow-up data for all participants. All data until the point of withdrawal from the trial are recorded.

Trial participants, care providers, the study team (including the study coordinator, trained raters) and the chief investigator are blinded to each participant’s treatment allocation. The data analyst and clinical pharmacist are unblinded to the treatment allocation. Unblinding of all participants may take place prior to the end of the study on safety grounds, in the case of a serious adverse event or events. This would be initiated by the Trial Steering Committee, in discussion with the chief pharmacist and the Data Monitoring Committee. In a medical emergency, the chief investigator or delegated clinician may unblind individual participants for their own safety and for further medical care.

#### Data collection plan

The primary outcome measure is the 11-item version of the ADAS-Cog used to measure cognition in patients with mild to moderate AD. It is considered the gold standard for assessing the efficacy of treatment in dementia clinical trials. The testing kit contains the study instruments with three sets of word lists for word recall and word recognition to be completed at week 0, week 4 and week 12 assessments.

The secondary outcome measures include the following:

Tests of Attention: Trails A and B is a validated timed test of complex attention. Trails Making Test Part A consists of 25 circles on a piece of paper with the numbers 1–25 written in random positions. It requires the subject to connect the circles in numeric order as quickly as possible within 90 s. Trails Making Test Part B consists of 25 circles on a piece of paper with the numbers 1–13 and the letters A–L written in random positions. It requires the subject to connect the circles in numeric and alphabetical order as quickly as possible, alternating between numbers and letters within 180 s. The trails are completed on worksheets with a pencil.

Digit-symbol substitution is a timed neuropsychological test sensitive to dementia. It consists of a key of nine digit-symbol pairs shown at the top of the page, followed by rows of numbers below missing symbols. It requires the subject to match symbols to numbers according to the given key as fast as possible. This is scored as the number of correct symbols within 60 s. The test is completed on a worksheet with a pencil.

Test of Everyday Attention (Map Search and Elevator Counting with Distraction) is a validated test of attention performed using everyday materials; the complete kit can be obtained through Pearsons including all materials and answer sheets [42]. In addition to the kit, the testing requires two coloured erasable markers (red and blue) and a CD player. The map search is a measure of visual selective attention and involves looking at a large map of Philadelphia in which the subject searches for a symbol within 2 min. Elevator counting with distraction is a measure of auditory selective attention, and it requires the subject to imagine they are in an elevator and listen to a series of tones to establish which floor the elevator has arrived at with the presence of distractor tones. There are three sets of symbols (A, B and C) and three CDs (A, B and C) to be completed at week 0, week 4 and week 12 assessments.

CANTAB-RVP (Rapid Visual Information Processing) [43] is a validated computerised measure of sustained attention administered in 7 min using an iPad tablet. The subject is required to look for a sequence of numbers; when they see the target sequence, they must respond by selecting the button in the centre of the screen as quickly as possible. To ensure that the subject can complete the RVP, they initially complete the CANTAB-MOT (Motor Screening Task), a 2-min task that screens for any sensorimotor deficits or lack of comprehension by asking the subject to select a coloured cross that appears in locations on the screen.

Carer Input and Activities of Daily Living: Neuropsychiatric Inventory (NPI) is a brief interview with the study partner to evaluate 12 behavioural areas commonly affected in patients with dementia; it is routinely used to evaluate the effects of treatment on these symptoms. Zarit Burden Interview (22 items) is a well-known measure of caregiver burden in caregivers of patients with dementia. It is a 22-item interview with the study partner. Each item on the interview is a statement which the subject is asked to answer on a 5-point scale. Alzheimer’s Disease Co-operative Study – Activities of Daily Living Inventory (ADCS-ADL) is commonly used to assess the performance of AD patients in 23 basic and instrumental activities of daily living (ADLs) giving a score from 0 to 78. It is completed with the study partner as a structured interview asking the subject to consider the past 4 weeks at the time of rating.

Toxicity and tolerability measurements including blood pressure and heart rate measurements are taken when the participants are sitting and after 1 min standing. Epworth Sleepiness Scale is widely used as a subjective measure of a subject’s sleepiness. It is a list of eight situations in which an individual rates their tendency to become sleepy on a 4-point scale giving a score from 0 to 24. The scale estimates whether the subject is experiencing excessive sleepiness and is completed with the patient and study partner. Common side effects of guanfacine (dry mouth, sedation, fatigue) are recorded using a patient and study partner reported Side Effects Questionnaire (SEQ). The participants are asked to report any side effects beginning with an open question addressing any new problems to then ask of five potential known side effects and a final question to ask of any new symptoms given a further five examples.

Observation of change following cessation of treatment as measured by Alzheimer’s Disease Co-operative Study – Clinical Global Impression of Change (ADCS-CGIC) [44]. Clinical Global Impression of Change (CGIC) is a measure of clinically meaningful change; it is a semi-structured interview based on the impression of the change completed with the patient and the study partner, using a modified version of the ADCS-CGIC with permission from the NIA Alzheimer’s Disease Cooperative Study (NIA Grant AG10483) to record the impression of change having ceased treatment.

The outcome measures are administered by a trained rater. Testing kits, study instruments and data collection forms are stored at the trial site. Where possible, to maximise standardisation and maintain consistency across administrations, the same trained rater will conduct the assessment in a similar testing environment at around the same time of day.

### Data management

The NorAD data management plan is consistent with the ICH Guideline for Good Clinical Practice (GCP; document E6 R2). Clinical data is entered into paper-based case report forms (CRFs) and then transferred into computers via InForm 6.0 (Oracle Health Sciences 6.0.1). The trained rater is given an InForm “data entry” account to enter data into the database. The study coordinator is given an InForm “coordinator” account to check data entered to ensure accuracy. Participants eligible for study entry are given a unique, sequential NorAD study identifier. At each assessment, data is entered onto CRFs and input into the study database. The data will be backed up electronically and securely stored locally. The files are backed-up onto a password-protected environment on a weekly basis. Hard copies will be stored locally, compliant with the Data Protection Act (2018).

### Data analysis

**Fig. 1:**
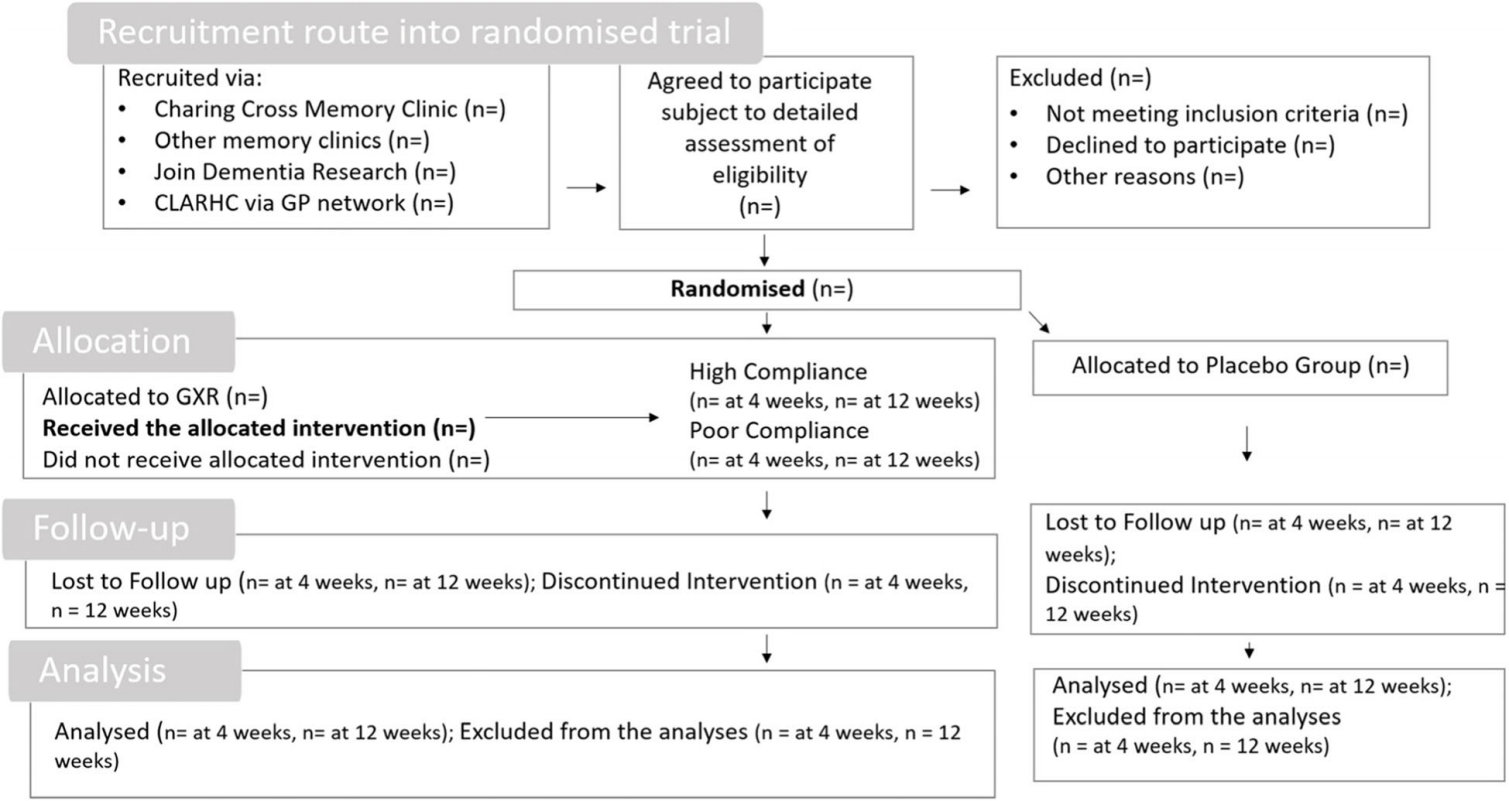
Consolidated Standards of Reporting Trials (CONSORT) guidelines

The data will be analysed and presented in accordance with the Consolidated Standards of Reporting Trials (CONSORT) guidelines in Fig. 1.

The primary analysis will be based on the intention-to-treat population. Participants are analysed according to the group they are randomised to, regardless of the treatment received. It will involve all available data, including the data from withdrawn participants and all levels of treatment compliance.

A secondary efficacy analysis will be based on the Complier Average Causal Effect (CACE) analysis, to evaluate the effect of taking guanfacine in practice (rather than the effect of being randomised to the treatment arm). The presumption within this CACE analysis is that random allocation to the guanfacine treatment arm, when not compliant with treatment, would result in the outcome that would have been observed, had they been allocated to and received placebo treatment. Non-compliance is defined as taking less than 5 doses per week on average in the interval between visits ending at the visit when ADAS-Cog is measured for use in the final analysis; they are also required to take at least 3 doses in the final week prior to ADAS-Cog assessment if their follow-up time is 13 weeks or more. This is based on the belief that the effect of GXR is likely to be short-acting symptomatic relief only.

The primary outcome measure is the ADAS-Cog at 12 weeks follow-up.

#### Missing data on individual questions which make up measurement scales

We will use the manuals on each questionnaire to determine the appropriate treatment of missing data items. When there is no information on this in the manuals, then we will replace the missing values by scaling up the outcome scale score according to the total possible scores from the missing and non-missing items on the questionnaire (providing the missing data does not account for more than 40% of the total for the specific patient).

#### Method of statistical analysis

All statistical models with numeric/questionnaire outcomes at follow-up will be analysed using mixed linear regression. There will be a separate model for each outcome variable, including one for ADAS-Cog. Outcome measurements taken at 4 weeks and at 12 weeks will form the mixed linear regression outcome variable, which will include a random intercept for each participant [45]. The regression model will be adjusted for a baseline measurement of the same outcome variable and for the concomitant medication (indicator variables for rivastigmine and galantamine use, rather than donepezil use, will be included), with indicator variables for time (4 weeks follow-up versus 12 weeks) and for the treatment group, and with an interaction between treatment and time (to allow for reporting of separate treatment effects at 4 weeks and at 12 weeks). Analyses will be further adjusted for severity of Alzheimer’s disease (mild or moderate, according to baseline MMSE score, as used in the stratified randomisation) and with indicator variables for the centre (if there is more than one full centre in the trial). All variables will be fitted as fixed effects initially. Time will be fitted as a random effects variable if the fit of the fixed effects model is poor (if *p* < 0.01 for improvement in fit from likelihood ratio model for primary outcome analysis, with the intention of reporting consistent models for all outcomes). For assessing the 4-week outcome, the indicator variable for time will be recoded as 1 for 12 weeks and 0 for 4 weeks. Log transformations will be applied, where appropriate, in order to render the outcome distributions closer to the normal. Boot-strap techniques will be undertaken to calculate the confidence intervals if neither the variable nor its log results in residuals from the regression analysis close to the normal distribution.

We will use multiple imputation to take account of any missing baseline data and follow-up data (when 4-week and 12-week data are both missing). Imputed values will be based on ADAS-Cog at baseline, on treatment group, and measurements within the same family (at available time points). MMSE and ADAS-COG are both in the “measuring severity of dementia” family, measures of attention form the “attention” family and measures of carer burden form another family, and measures of patient competency and behaviour form the final family. If fewer than 20% of patients have missing outcome data at either baseline or at both 4 weeks and 12 weeks follow-up (or fewer than 10% for ADAS-Cog and for MMSE), then reported results will be based on complete case analysis, on the basis that the multiple imputation results would be very similar in this situation.

A full, detailed statistical analysis plan has been written and signed. Deviations from this plan will be reported in the final analysis and report.

Patient demographics and other baseline information will be summarised by treatment group. Continuous variables following a normal distribution will be summarised using means (and standard deviation). Highly skewed variables will be summarised using medians (interquartile or full range). Categorical variables (binary and ordinal and multinomial) will be presented as frequencies and percentages. Descriptive analyses, including histograms, normal probability plots and box plots, will be used to assess the distributional assumptions and check for possible outliers.

Serious adverse events and any adverse events (occurring from 0 to 13 weeks) considered to be relevant will be presented. The number of participants who suffer at least one of each event type will be compared between the GXR group (those who received at least one dose) and those allocated to placebo (who did not receive any GXR) using Fisher’s exact test. The results will be interpreted taking account of multiple comparisons made.

### Trial oversight

While the chief investigator has overall responsibility for the conduct of the study, the Trial Management Group (composed of clinical and non-clinical researchers) has responsibility for the day-to-day management of the trial. The study is subject to inspection and audit by Imperial College London as the sponsor and study coordination centre. This includes regular on-site monitoring to ensure adherence to Good Clinical Practice (GCP). The Trial Steering Committee (TSC) acts as the oversight body for the trial with responsibilities including assuring study integrity and design. This TSC committee includes the chief investigator, the trial statistician and members independent of the investigators, employing organisations, funders and sponsors. The committee oversees the management of the trial, the progress and conduct along with advising on scientific credibility. This TSC committee considers and acts as appropriate upon the recommendations of the Data Monitoring Committee (DMC). This DMC committee comprises clinicians and an external statistician to ensure the trial continues for an adequate period to answer its scientific questions alongside monitoring participant safety. Both committees are convened at least three times during the trial: after the recruitment of 10 patients, after the recruitment of 40 patients and after 80 patients have completed the study. There is no formal interim analysis for efficacy planned for NorAD.

### Current trial status

Recruitment is ongoing. As of 01 March 2021, 63 patients have been enrolled with 56 having completed the trial. There have been 2 patients withdrawn due to an SAE and a further 2 whose participation was suspended during COVID-19 and are now considered withdrawn. The current end date for the trial is 31 December 2022.

## Discussion

The NorAD trial is a symptomatic trial examining the effects of a noradrenergic alpha-2A agonist, guanfacine, in combination with standard anticholinesterase inhibitors, on cognition and attention in patients with mild to moderate Alzheimer’s disease.

In conducting the study, we have experienced some practical and operational issues. We have had to exclude a number of individuals for not having an available study partner even though the patient themselves was interested in participating. As a single-site study, most of our participants are based in London, but we have received referrals for patients based over 100 miles away. Where possible, we have tried to include these participants and have arranged transport.

Although the trial was suspended as part of a general suspension of clinical research at the beginning of the UK lockdown in March 2020, it was restarted with an amended protocol in August 2020 and is ongoing. If the analysis shows guanfacine to be superior to placebo, then this medication, which is already licenced for the treatment of ADHD, will be a significant addition to therapeutic options in symptomatic Alzheimer’s disease.

## Appendix 1 List of contradictions to guanfacine as per the SMPC

The following drugs should not be commenced during the trial:

Ketoconazole, rifampicin, sodium valproate, beta blockers, non-dihydropyridine calcium channel blockers, cholinesterase inhibitors and sphingosine-1 phosphate receptor modulators

The following drugs, which can increase the QT interval, should not be commenced during the trial:

Class IA antiarrhythmics (e.g. quinidine, procainamide, disopyramide), class III antiarrhythmics (e.g. amiodarone, sotalol, ibutilide, dronedarone), class 1C antiarrhythmics (e.g. flecainide, propafenone), antipsychotics (e.g. chlorpromazine, pimozide, haloperidol, droperidol, ziprasidone), antidepressants (e.g. fluoxetine, citalopram, venlafaxine, tricyclic/tetracyclic antidepressants, e.g. amitriptyline, imipramine, maprotiline), opioids (e.g. methadone), macrolide antibiotics and analogues (e.g. erythromycin, clarithromycin, telithromycin, tacrolimus), quinolone antibiotics (e.g. moxifloxacin, levofloxacin, ciprofloxacin), antimalarials (e.g. quinine, chloroquine), azole antifungals (e.g. ketoconazole, fluconazole, voriconazole), domperidone, 5-HT3 receptor antagonists (e.g. dolasetron, ondansetron), tyrosine kinase inhibitors (e.g. sunitinib, nilotinib, lapatinib), histone deacetylase inhibitors (e.g. vorinostat) and beta-2 adrenoceptor agonists (e.g. salmeterol, formoterol)

To minimise the possible sedative side effects of GXR, the following central nervous system depressants should not be commenced during the trial:

Sedatives, hypnotics, benzodiazepines, barbiturates, antipsychotics and oral methylphenidate

## Abbreviations

AChEIs: Acetylcholinesterase inhibitors
AD: Alzheimer’s disease
ADAS: Alzheimer’s Disease Assessment Scale
ADAS-Cog: Alzheimer’s Disease Assessment Scale-Cognitive Subscale
ADCS-ADL: Alzheimer’s Disease Co-operative Study – Activities of Daily Living Inventory
ADCS-CGIC: Alzheimer’s Disease Co-operative Study – Clinical Global Impression of Change
ADHD: Attention deficit hyperactivity disorder
CANTAB-RVP: Cambridge Neuropsychological Automated Battery – Rapid Visual Information Processing
ChEI: Cholinesterase inhibitor
CRN: North West London Clinical Research Network
DSS: Digit-symbol substitution
ESS: Epworth Sleepiness Scale
GXR: Extended-release guanfacine
IMP: Investigative Medicinal Product
JDR: NIHR Join Dementia Research Network
MMSE: Mini-Mental State Examination
NINCDS-ADRDA: National Institute of Neurological and Communicative Disorder and Stroke and Alzheimer’s Disease and Related Disorders Association
NorAD: Noradrenergic Add-on Therapy with Extended-Release Guanfacine in Alzheimer’s Disease
NPI: Neuropsychiatric Inventory
PIC: Participant identification centres
SEQ: Side Effects Questionnaire
TEA: Tests of Everyday Attention
ZBI: Zarit Burden Interview
